# Combined Control for a Piezoelectric Actuator Using a Feed-Forward Neural Network and Feedback Integral Fast Terminal Sliding Mode Control

**DOI:** 10.3390/mi15060757

**Published:** 2024-06-05

**Authors:** Eneko Artetxe, Oscar Barambones, Isidro Calvo, Asier del Rio, Jokin Uralde

**Affiliations:** System Engineering and Automation Department, Faculty of Engineering of Vitoria-Gasteiz, Basque Country University (UPV/EHU), 01006 Vitoria-Gasteiz, Spain; isidro.calvo@ehu.eus (I.C.); asier.delrio@ehu.eus (A.d.R.); jokin.uralde@ehu.eus (J.U.)

**Keywords:** recurrent neural network, integral fast terminal sliding mode control, piezoelectric actuator, hysteresis

## Abstract

In recent years, there has been significant interest in incorporating micro-actuators into industrial environments; this interest is driven by advancements in fabrication methods. Piezoelectric actuators (PEAs) have emerged as vital components in various applications that require precise control and manipulation of mechanical systems. These actuators play a crucial role in the micro-positioning systems utilized in nanotechnology, microscopy, and semiconductor manufacturing; they enable extremely fine movements and adjustments and contribute to vibration control systems. More specifically, they are frequently used in precision positioning systems for optical components, mirrors, and lenses, and they enhance the accuracy of laser systems, telescopes, and image stabilization devices. Despite their numerous advantages, PEAs exhibit complex dynamics characterized by phenomena such as hysteresis, which can significantly impact accuracy and performance. The characterization of these non-linearities remains a challenge for PEA modeling. Recurrent artificial neural networks (ANNs) may simplify the modeling of the hysteresis dynamics for feed-forward compensation. To address these challenges, robust control strategies such as integral fast terminal sliding mode control (IFTSMC) have been proposed. Unlike traditional fast terminal sliding mode control methods, IFTSMC includes integral action to minimize steady-state errors, improving the tracking accuracy and disturbance rejection capabilities. However, accurate modeling of the non-linear dynamics of PEAs remains a challenge. In this study, we propose an ANN-based IFTSMC controller to address this issue and to enhance the precision and reliability of PEA positioning systems. We implement and validate the proposed controller in a real-time setup and compare its performance with that of a PID controller. The results obtained from real PEA experiments demonstrate the stability of the novel control structure, as corroborated by the theoretical analysis. Furthermore, experimental validation reveals a notable reduction in error compared to the PID controller.

## 1. Introduction

High-precision positioning was primarily limited to academic domains. However, advancements in fabrication methods since the turn of the century have opened up new possibilities for incorporating micro-actuators into industrial environments. Piezoelectric actuators (PEAs) have since become indispensable in numerous applications requiring precise control and manipulation of mechanical systems. They are integral components in the micro-positioning systems used in nanotechnology, microscopy, and semiconductor manufacturing and facilitate extremely fine movements and adjustments [1]. Additionally, PEAs are employed in precision positioning systems for optical components, mirrors, and lenses [2], enhancing the accuracy of laser systems, telescopes, and image stabilization devices [3]. They also play a vital role in vibration control systems by damping or controlling vibrations in mechanical structures and machinery to improve stability and reduce noise [4,5]. In the medical field, PEAs are utilized in devices such as micro-manipulators for minimally invasive surgery, ultrasound imaging equipment, and drug delivery systems [6]. Furthermore, in aerospace and defense applications, PEAs contribute to tasks such as fine-tuning flight control surfaces, actuation in precision instruments, and vibration isolation in sensitive equipment [7,8]. Overall, PEAs serve as essential components in mechatronic systems, including robotics, enabling precise positioning of robotic arms, grippers, and end-effectors and providing better control motion in various industrial domains [9].

PEAs offer several advantages, such as improved precision positioning, fast response times, and a wide range of operating frequencies. They are capable of producing large forces and displacements with relatively low power consumption, making them suitable for applications requiring high dynamic performance and energy efficiency. Moreover, piezoelectric materials exhibit inherent stiffness and mechanical stability, enabling precise control and positioning at micrometer and nanometer scales [10]. Additionally, they are compact, lightweight, and resistant to electromagnetic interference, making them suitable for use in harsh environments and confined spaces. Unfortunately, PEAs present some limitations. They typically operate over limited displacement ranges and exhibit nonlinear behavior due to certain phenomena such as hysteresis [11,12], creep [13], and temperature sensitivity [14]. Furthermore, they require high driving voltages and sophisticated control electronics, which can increase system complexity and cost [15]. Additionally, piezoelectric materials are brittle and can be susceptible to fatigue and degradation over time, particularly under cyclic loading conditions [16]. Despite these challenges, advances in material science, manufacturing techniques, and control strategies continue to expand the capabilities and applications of PEAs. Hysteresis is a crucial and extensively studied phenomenon, particularly in systems where high precision is essential, such as guidance systems. It can introduce errors of up to 22% [17], significantly impacting accuracy. Moreover, hysteresis cannot be overlooked as it not only affects achieving the desired position but can also lead to system instability [18].

Hysteresis in PEAs is a complex phenomenon resulting from the interaction between mechanical strain and an electric field. When subjected to an electric field, the polarization of the material’s domains becomes aligned in an arbitrary direction [19]. As the electric field increases, the poles of the material align with the field, resulting in elongation due to the ferroelectric effect. When the electric field decreases, the poles attempt to return to their initial orientations but with a certain deviation from their original positions [20]. This difference between the initial and final states of the polarization creates a hysteresis loop, which manifests as a lag in the actuator’s response to changes in the applied voltage or force. Addressing hysteresis is essential for enhancing the accuracy and reliability of piezoelectric-based systems in different applications [21].

Hysteresis in PEAs can be reduced through various techniques and approaches [22]. One approach involves improving the design and fabrication of the piezoelectric material itself to minimize hysteresis. This may include optimizing the material composition, structure, and processing techniques to achieve more uniform polarization behavior. Another common method is to implement compensation algorithms in the control system to account for the non-linearity caused by hysteresis. These algorithms can use mathematical models or experimental data to predict and counteract the hysteresis effect in real time.

To mathematically model hysteresis behavior, several models have been developed. The Preisach model [23] represents hysteresis using a distribution of hysterons, while the Jiles–Atherton [24] model adapts concepts from magnetic materials to describe piezoelectric hysteresis. The Bouc–Wen model, commonly used in structural engineering, captures non-linear hysteresis through a set of differential equations [25]. Additionally, the Prandtl–Ishlinskii model decomposes hysteresis into elementary operators, allowing for accurate representation of a wide range of hysteresis behaviors [26]. Each model offers unique advantages and complexity levels, enabling engineers to select the most appropriate model based on application requirements and experimental data.

Machine learning techniques have become increasingly prevalent for modeling the hysteresis behavior of PEAs from experimental data. Among the principal machine learning approaches for this purpose are artificial neural networks (ANNs) [27,28,29], support vector machines (SVMs) [30], random forests [31], and Gaussian processes (GPs) [32]. These machine learning techniques provide flexible and data-driven approaches to hysteresis modeling, enabling accurate predictions and enhanced understanding of the dynamic characteristics of PEAs [27]. Recurrent artificial neural networks (RNNs) have become a powerful tool for modeling non-linear dynamics [33] such as PEA hysteresis behavior. Unlike feed-forward neural networks, RNNs have feedback connections that allow them to retain information about previous states, making them adequate for modeling dynamic systems with temporal dependencies, such as hysteresis phenomena. By incorporating feedback loops, RNNs can capture the temporal dynamics of the input–output relationship in PEA systems, enabling them to effectively model hysteresis loops over time.

The rate dependency of PEA hysteresis is a critical factor in its performance, particularly when operating across a wide range of frequencies. Traditional hysteresis models are often rate-independent and fail to accurately capture the dynamic behavior of PEAs under varying conditions. This dependency manifests as changes in the width of the hysteresis loop and the amplitude of displacement response, especially at higher frequencies.

Laboratory experiments have been performed to characterize the rate-dependent hysteresis properties of a piezoceramic actuator under various excitations in the 0.1–500 Hz frequency range and have revealed larger hysteresis loop widths and lower displacement response amplitudes above 10 Hz. A rate-dependent Prandtl–Ishlinskii model has been developed and validated and showed very good agreement with experimental data across different input types and frequencies [34]. The paper models the rate-dependent behavior of piezoelectric materials using a three-dimensional finite element framework by applying a rate-dependent polarization framework to cyclic electrical loading at various frequencies. The onset of domain switching is determined by the reduction in free energy, and intergranular effects are captured via a probabilistic approach. Numerical simulations for PIC-151 ceramics show good agreement with experimental electric displacement versus electric field data [35]. This paper presents a novel modified inverse Preisach model to compensate for the rate-dependent hysteresis of a piezoelectric actuator across varying frequency ranges. By adopting the fast Fourier transform method to select proper μ-density functions and weights, the proposed model forms a real-time online rate-dependent compensator, significantly improving the tracking control accuracy of the actuator [36].

Robust controllers ensure stability and performance in control systems amidst uncertainties and disturbances. These controllers are designed to maintain desired system behavior even in the presence of variations or unknown factors. The use of robust control techniques systems can effectively mitigate external disturbances and uncertainties, thus enhancing their resilience and reliability. Robust controllers have been explored for PEA positioning with proven results. A robust control scheme based on inverse models and accompanied by a stability analysis is presented in [16]. This approach yielded favorable outcomes, achieving an error of approximately 0.5 μm. Several first-order sliding mode control (SMC) approaches have been devised and employed for PEAs, despite the significant drawback posed by chattering [37,38]. Integral sliding mode control (ISMC), introduced in [39], offers another strategy for mitigating static error. However, its practicality diminishes when a mathematical model is employed due to the complexities of the dynamics, leading to reduced control accuracy [40]. Integral fast terminal SMC (IFTSMC) controllers have proven their robustness, fast convergence, and ability to handle uncertainties and disturbances effectively. The addition of integral action to fast terminal SMCs ensures that any steady-state errors are minimized. This integral action helps to improve the tracking accuracy and disturbance rejection capabilities of the controller. The IFTSMC scheme has been proven in different domains, with promising results [41,42,43].

This work proposes an ANN-based IFTSMC controller for piezoelectric actuator positioning. The ANN solves the necessity of having an accurate model of the non-linear dynamics of PEAs. The proposed controller is implemented in a real-time setup to validate the proposed solution. The performance of the PEA IFTSMC controller is compared to that of a PID controller since PID controllers are frequently used in the literature as reference controllers [44,45].

The paper is structured as follows: Section 2 presents and discusses the hardware utilized and provides a brief overview of the hysteresis phenomenon. Furthermore, it introduces the ANN-based IFTSMC: with subsections dedicated to the discontinuous terms and the ANN. Additionally, a Lyapunov analysis is included to demonstrate the stability of the system with this novel controller. Finally, Section 3 evaluates the real-time performance of the ANN: presenting results and comparisons between the PI controller and the ANN-based IFTSMC. Finally, Section 4 provides the conclusion: summarizing the findings and discussing the implications of the study.

## 2. Materials and Methods

### 2.1. Hardware

This study utilizes commercial hardware from Thorlabs: specifically, the PK4FYC2 stack actuator. This actuator comprises multiple piezoelectric chips bonded together with epoxy and glass beads. It operates on a micrometric scale, and its elongation is measured using four strain gauges arranged in a Wheatstone bridge configuration. The actuator accepts voltage inputs ranging from 0 to 150 V, with 150 V resulting in the maximum displacement of 38.5 μm. The manufacturer specifies a maximum error of 15%, primarily attributed to hysteresis, which can be mitigated by implementing a PID controller within a feedback control system. Additional technical specifications are provided below.

The voltage range of 0–150 V is generated using a single-channel driver cube, Thorlabs KPZ101, which is specifically recommended for the PEA. This driver cube is versatile and is compatible with a wide range of actuators. It offers convenient operation in open-loop mode without requiring a peripheral computer. Additionally, it can function in closed-loop mode with an external 0–10 V signal, scaling it up to 0–150 V for the PEA, with a maximum allowed bandwidth of 1 kHz.

Due to the Wheatstone-bridge-based measurement method, the elongation is represented as a resistance change, which can be challenging to read due to the small values involved. To address this issue, the manufacturer recommends using the pre-amplifier AMP002. This pre-amplifier amplifies the small differences in a 0–2 V signal, which is then fed into a cube reader: the Thorlabs KSG101. This reader displays the PEA extension on an embedded LED viewer and provides an output signal ranging from 0 to 10 V. Table 1 provides the technical details of the PEA elements.

As mentioned earlier, both the driving and measurement signals operate within a 0–10 V range. Therefore, a dSpace DS1104 board is utilized for acquisition and control purposes. This hardware is equipped with real-time interface (RTI) capabilities, reducing compilation time for driving algorithms and enabling real-time control tuning. The board is connected via a peripheral component interconnect (PCI) bus to a Dell Precision Workstation T3500 (Warszawa, Poland) featuring an Intel 64 2.4 GHz microprocessor and 18 GB of available memory.

**Figure 1:**
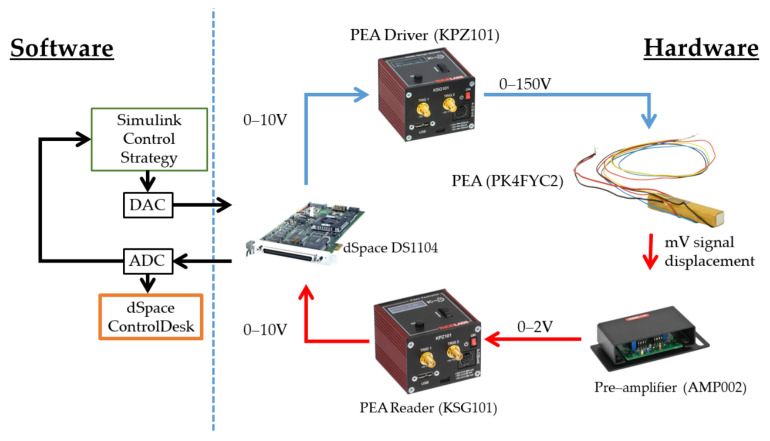
Scheme of the software and hardware implementation.

The control architecture is exclusively designed in Simulink 2022B by MathWorks and is implemented through dSpace RTI. This architecture is designed with flexibility for real-time operation and facilitates gain tuning and performance metric calculation. Real-time visualization of data is achieved using ControlDesk 2022B, while MATLAB by MathWorks is utilized for data processing and visualization. A sampling time of 1 kHz is established for all experiments, aligning with the relationship between data acquisition and hardware physical limitations. Figure 1 provides a schematic overview of the flow between the hardware and software components.

### 2.2. Hysteresis

PEA hysteresis refers to the phenomenon observed in PEAs whereby the displacement of the actuator depends not only on the current input voltage but also on its previous history. This behavior arises due to the inherent properties of the material used in PEAs, which exhibit hysteresis characteristics. Hysteresis in PEAs occurs because the material undergoes irreversible changes in its internal structure when subjected to an electric field, resulting in a lag or memory effect in the actuator’s response. Specifically, when the input voltage is increased or decreased, the actuator’s displacement may not immediately follow the input signal due to this hysteresis effect. Instead, the displacement may lag or exhibit a different trajectory depending on the previous history of the input voltage. This hysteresis behavior can complicate the control of PEAs as it introduces non-linearities and memory effects that must be accounted for in the control algorithm. Therefore, understanding and modeling PEA hysteresis is essential for designing effective control strategies to accurately predict and control the actuator’s behavior.

The hysteresis cycle in PEAs refers to the characteristic loop-shaped behavior observed when plotting the actuator’s displacement against the applied voltage. This cycle typically consists of two main phases: the loading phase and the unloading phase.

During the loading phase, as the voltage applied to the PEA increases, the actuator undergoes a corresponding displacement, typically in the positive direction. This displacement is non-linear and may exhibit gradual increases or sudden jumps depending on factors such as the voltage magnitude and rate of change. As the voltage continues to increase, the actuator reaches its maximum displacement, which corresponds to the maximum voltage applied.

**Figure 2:**
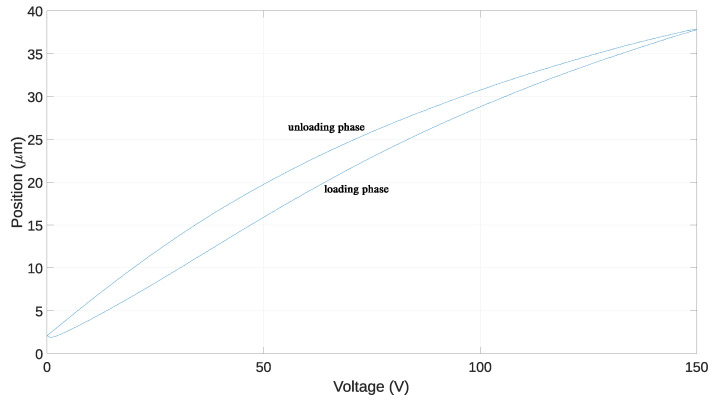
Hysteresis through a complete cycle.

In the unloading phase, when the voltage is decreased, the actuator begins to retract or return to its original position. However, due to hysteresis effects, the actuator’s displacement may not immediately decrease in proportion to the voltage reduction. Instead, the displacement lags behind the voltage change, resulting in a different trajectory compared to the loading phase. This lag or memory effect is characteristic of hysteresis and is a key factor that complicates the control of PEAs. Figure 2 shows the hysteresis of a PEA when a 1 Hz triangular signal is applied. The different curves correspond to the loading phase and the unloading phase.

### 2.3. Control Design and Performance Metrics

**Figure 3:**
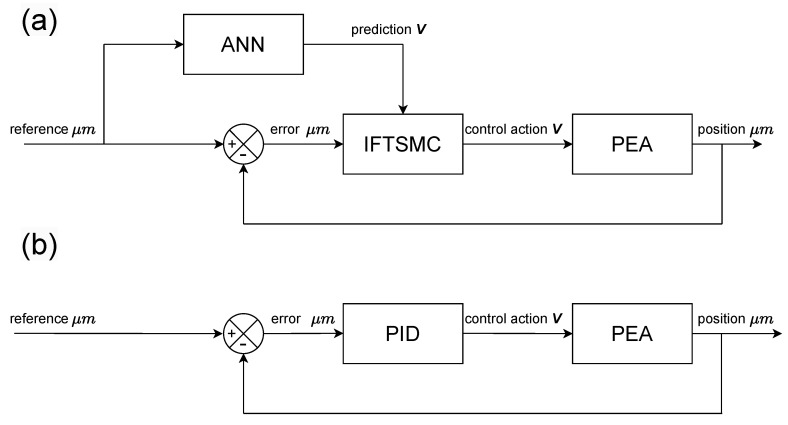
Control schemes for (**a**) the proposed ANN-based IFTSMC and (**b**) the PID controller.

In this research, the focus was on implementing and evaluating two distinct control architectures through practical experimentation to ascertain their efficacy and performance in real-world scenarios. The experimental setups were designed to embody these architectures and provide a tangible platform for analysis and comparison. Figure 3 illustrates a schematic representation of these structures. To optimize the control process, the controllers were endowed with specific degrees of freedom, which were primarily associated with tuning constants that were crucial for their operation. The tuning process involved iteratively adjusting these constants to achieve the desired control outcomes. A key aspect of this tuning methodology was the utilization of the integral of the absolute error (IAE) reduction technique. This approach aims to minimize the error between the desired and actual control responses by optimizing the controller’s parameters. In Equation (1), the first term represents the expression for the IAE, which serves as the objective function for parameter optimization.

In addition to the IAE, which serves as a pivotal metric for evaluating guidance performance, this study incorporates two supplementary metrics: the root-mean-square error (RMSE) and the relative root-mean-square error (RRMSE). These additional metrics, inspired by the methodology outlined by the authors of reference [46], provide nuanced insights into the control system’s efficacy by quantifying the magnitude and relative significance of deviations between observed and desired outcomes. The RMSE, calculated as the square root of the average of the squared differences between actual and reference values across all samples, offers a measure of the overall accuracy of the control system. On the other hand, the RRMSE normalizes the RMSE against the range of the reference values, providing a relative measure of error that facilitates comparison across different datasets. By integrating these supplementary metrics alongside the IAE, this research aimed to furnish a comprehensive assessment of the control system’s performance, enabling a nuanced understanding of its effectiveness at meeting desired guidance objectives.

(1) $$\left\{\begin{array}{c} IAE=\sum_{i=1}^{N}\left|e_{i}\right|\Delta t\\ IAEmean=\frac{\sum_{i=1}^{N}\left|e_{i}\right|\Delta t}{N}\\ RMSE=\sqrt{\frac{1}{N}\sum_{i=1}^{N}e_{i}^{2}}\\ RRMSE=100\cdot \sqrt{\sum_{i=1}^{N}e_{i}^{2}/\sum_{i=1}^{N}r_{i}} \end{array}\right.$$

### 2.4. PI Control

Proportional–integral (PI) control is a widely used method in control engineering and is renowned for its simplicity and effectiveness at regulating various systems across diverse industries. Rooted in the principles of feedback control, a PI controller combines proportional and integral actions to achieve desired system performance.

Proportional control provides control action that is proportional to the current error, allowing the system to respond to deviations from the set point. However, it may lead to steady-state errors if there are inherent system biases. Integral control is designed to eliminate steady-state errors by continuously integrating the error signal over time and adding it to the control signal. The integral action acts to reduce the accumulated error over time. The aim of control is to keep the actuator within the reference position. Thus, the error is defined as the difference between the real position and the reference position, as shown by Equation (2). By combining proportional and integral actions, PI control provides a balance between responsiveness and stability, making it suitable for a wide range of control applications. The control function *u* of the PI controller is shown in Equation (3):

(2) $$e_{r}=x-x_{ref}$$

(3) $$u=k_{p}\;e_{r}+k_{i}\int_{0}^{t}e_{r}\cdot dt$$

where $e_{r}$ is the error, *x* is the measured position, $x_{ref}$ is the reference position, *u* is the control action, and $k_{p}$ and $k_{i}$ are the proportional and integral gain coefficients, respectively. The PI controller faces challenges in determining optimal proportional and integral gain coefficients and is susceptible to variations in system load. Tuning $k_{p}$ and $k_{i}$ requires understanding system dynamics and often involves trial and error or advanced methods. Load variations can lead to deviations from desired set points, causing oscillations or slow responses. Despite challenges, the PI controller remains popular due to its simplicity and effectiveness in various industrial applications.

### 2.5. ANN

The challenges faced by traditional PEA models in accurately representing system dynamics are multifaceted. One of the primary issues arises from the presence of asymmetric effects, which can introduce non-linearities into the system’s behavior. Additionally, the complexity of model implementation often necessitates sophisticated mathematical formulations, leading to high computational demands. As a result, conventional PEA models may struggle to adequately capture the intricacies of system dynamics, particularly when confronted with real-world complexities.

To address these challenges, an alternative approach leveraging artificial neural networks (ANNs) has emerged. ANNs offer a powerful tool for learning complex non-linear relationships directly from data, making them well-suited for modeling systems with hysteresis and other non-linear phenomena. By integrating an ANN into the control framework, the linearity and hysteresis dynamics can be effectively compensated for, enhancing the accuracy and robustness of the model. The ANN adaptively compensates the control signal based on the system’s current state and past history. By learning from the system’s behavior, the ANN can effectively compensate for hysteresis and non-linearities, improving the overall performance of the control system.

Identifying hysteresis using artificial neural networks (ANNs) typically involves architectures capable of capturing non-linear relationships and memory effects. Recurrent neural networks (RNNs)—particularly, long short-term memory (LSTM) networks—are often suitable for this task due to their ability to retain information over time. LSTM networks can effectively model temporal dependencies, making them suitable for capturing hysteresis behavior, which involves the system’s response being influenced by its historical states. Additionally, gated recurrent unit (GRU) networks, a variant of RNNs similar to LSTMs, can also be used for hysteresis identification. GRUs have a simpler architecture compared to LSTMs but still possess memory capabilities that allow them to capture temporal dependencies effectively. Overall, RNN architectures like LSTMs and GRUs are commonly preferred for identifying hysteresis due to their ability to model complex temporal relationships, making them suitable for tasks involving memory and non-linear dynamics.

**Figure 4:**
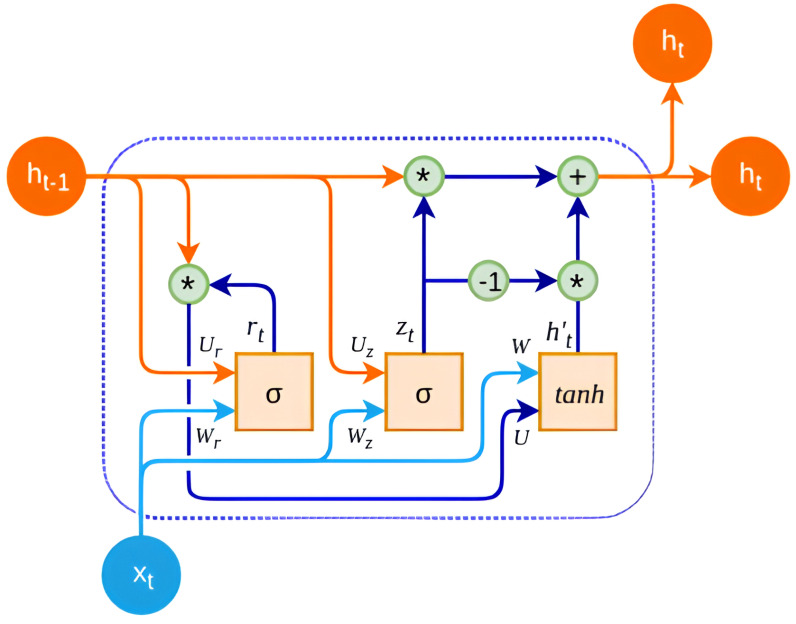
GRU layer information flow diagram.

For this research a GRU-based RNN was selected due to its ability to effectively model temporal dependencies in the data while mitigating issues like over-fitting and vanishing gradients. Additionally, GRUs have been shown to perform well in various sequence modeling tasks, including non-linear modeling, natural language processing, and time series prediction. The GRU-based LRNN structure has four layers formed by the input layer, GRU layer, as shown in Figure 4, fully connected layer, and an output regression layer. GRUs contain gating mechanisms that control the flow of information within the network, allowing them to capture and retain relevant information over longer sequences. The key components of a GRU layer include the update gate (z), which determines how much of the previous hidden state to retain and how much of the new candidate activation to add to the hidden state; the reset gate (r), which controls how much of the previous hidden state to forget when computing the new candidate activation; the candidate activation, which is new memory content computed based on the input at the current time step and the previous hidden state and serves as a candidate for updating the current hidden state; and the hidden state (h), representing the memory of the network at each time step and updated based on the candidate activation controlled by the update gate.

### 2.6. ANN-Based IFTSMC

IFTSMC, or intermittent feedback terminal sliding mode control, represents a novel approach to terminal sliding mode control developed by Venkataraman and Gulati [47] at the Jet Propulsion Laboratory. Unlike traditional sliding mode control techniques, IFTSMC is characterized by its non-linearity and robustness. This innovative approach combines the advantages of integral control and terminal sliding mode control and offers robust performance, finite-time convergence, and enhanced tracking accuracy.

The integration of integral action ensures steady-state precision by eliminating steady-state error, while the terminal sliding mode component enables rapid convergence to the desired trajectory, even in the presence of uncertainties and disturbances. This unique combination makes IFTSMC well-suited for a wide range of applications, including aerospace, robotics, power systems, and renewable energy.

The control law $u_{c}$ in IFTSMC comprises two distinct terms: a discontinuous term $u_{sw}$, which is responsible for maintaining the system on the sliding surface, and an equivalent term $u_{eq}$, which is designed to drive the system towards the sliding surface [48]. The combined command law, expressed by Equations (5) and (6), encapsulates the control action necessary for reducing the tracking error in Equation (4):

(4) $$e_{r}=x-x_{ref}$$

(5) $$u_{c}=u_{ann}+u_{smc}$$

(6) $$u_{smc}=u_{eq}+u_{sw}$$

where $u_{ann}$ represents the output of the ANN, and the term $u_{smc}$ comprises both the equivalent and the discontinuous components.

The discontinuous term $u_{sw}$ is defined in Equation (7), while calculation of the equivalent term is done later on in Equation (19).

(7) $$u_{sw}=-K\cdot sign\left(s\right)$$

where *s* is the sliding surface, and K must be a positive constant.

The sliding surface *s* is represented in Equation (8):

(8) $$s={\dot{e}}_{r}+\alpha e_{r}+\lambda {\left(\int_{0}^{t}e_{r}\;dt\right)}^{\frac{p}{q}}$$

where *t* is the time at the moment, and α, λ, *p*, and *q* are positive constants, with $1<\frac{p}{q}<2$ being satisfied.

#### Stability Proof

The PEA is considered as a second-order mechanical system [49] as in Equation (9):

(9) $$m\ddot{x}+b\dot{x}+kx+d\;f_{h}\left(x\right)=d\;u+P$$

where *m* is the mass, *b* is the damping, *k* is the stiffness, *x* is the position, *d* is the piezoelectric coefficient, $f_{h}\left(x\right)$ is the hysteresis, *u* is the input voltage, and *P* is the uncertainties, unmodeled dynamics, and perturbations. The piezoelectric coefficient *d* is defined as the product of the stiffness and the maximum displacement divided by the maximum driving voltage.

The control signal is designated by Equation (5). The term $u_{ann}$ contains a linear part and the hysteresis, so it can be decomposed as in Equation (10):

(10) $$u_{ann}=u_{linear}+f_{ann}\left(x\right)$$

The term $f_{ann}\left(x\right)$ is analogous to $f_{h}\left(x\right)$ in Equation (9). The term $u_{linear}$ can be defined as the mechanical system without taking into account hysteresis or perturbations, as in Equation (11):

(11) $$u_{linear}=\frac{1}{d}\left(m{\ddot{x}}_{ref}+b{\dot{x}}_{ref}+kx_{ref}\right)$$

where $x_{ref}$ is the reference position.

Replacing it in Equation (10) $u_{ann}$ gives Equation (12):

(12) $$u_{ann}=\frac{1}{d}\left(m{\ddot{x}}_{ref}+b{\dot{x}}_{ref}+kx_{ref}\right)+f_{ann}\left(x\right)$$

Combining the control signal from Equation (12) in Equation (9) gives Equation (13) as follows:

(13) $$m\ddot{x}+b\dot{x}+kx+d\;f_{h}\left(x\right)=m{\ddot{x}}_{ref}+b{\dot{x}}_{ref}+kx_{ref}+d\;f_{ann}\left(x\right)+d\;u+P$$

where $u=u_{eq}+u_{sw}$.

The error is defined as $e_{r}$ in Equation (4), and there will always be a difference between what the ANN predicts and the actual outcomes. This error is a natural part of the learning process for ANNs and arises due to various factors such as the complexity of the problem being tackled and the limitations of the network architecture. This error is defined with $\epsilon_{ann}$ in Equation (14) as the difference between the ANN’s prediction of the hysteresis and the actual hysteresis. This simplifies Equation (13) in Equation (15):

(14) $$\epsilon_{ann}=f_{ann}\left(x\right)-f_{h}\left(x\right)$$

(15) $$m{\ddot{e}}_{r}+b{\dot{e}}_{r}+ke_{r}=d\;\epsilon_{ann}+d\;u+P$$

From Equation (15), we can obtain the second derivative of the error in Equation (16):

(16) $${\ddot{e}}_{r}=-\frac{b}{m}{\dot{e}}_{r}-\frac{k}{m}e_{r}+\frac{d}{m}\;\epsilon_{ann}+\frac{d}{m}\;u+\frac{P}{m}$$

The sliding surface defined in Equation (8) is derived to obtain Equation (17). Then, Equation (16) is substituted in (17) to obtain Equation (18):

(17) $$\dot{s}=-\frac{b}{m}{\dot{e}}_{r}-\frac{k}{m}e_{r}+\frac{d}{m}\;\epsilon_{ann}+\frac{d}{m}\;u+\frac{P}{m}+\alpha \;{\dot{e}}_{r}+\lambda \;\frac{p}{q}\;e_{r}\;{\left(\int_{0}^{t}e_{r}\;dt\right)}^{\frac{p-q}{q}}$$

To simplify Equation (17), all the uncertainties are encapsulated under the term ρ:

(18) $$\rho =\frac{d}{m}\epsilon_{ann}+\frac{P}{m}$$

From Equation (17), we obtain the term $u_{eq}$ when $\dot{s}=0$, as shown in Equation (19):

(19) $$u_{eq}=\frac{b}{d}{\dot{e}}_{r}+\frac{k}{d}e_{r}-\frac{m}{d}\rho -u_{sw}-\frac{m}{d}\;\alpha \;{\dot{e}}_{r}-\frac{m}{d}\;\lambda \;\frac{p}{q}\;e_{r}\;{\left(\int_{0}^{t}e_{r}\;dt\right)}^{\frac{p-q}{q}}$$

The obtained control law can be analyzed with the Lyapunov theory of stability. If there exists a positive definite function $R^{n}\to R$ so that V(x)>0, V(0)=0, V(∞)=∞, and $\dot{V}\left(x\right)<0\forall x\neq 0$, the dynamical system is asymptotically stable. Therefore, a Lyapunov candidate function that satisfies the requirements is proposed in Equation (20):

(20) $$V\left(s\right)=\frac{1}{2}s^{2}$$

The derivative of the above Equation is expressed in Equation (21), and we substitute $\dot{S}$ from Equation (17):

(21) $$\dot{V}\left(s\right)=s\;\dot{s}=s\left(-\frac{b}{m}{\dot{e}}_{r}-\frac{k}{m}e_{r}+\frac{d}{m}\;\epsilon_{ann}+\frac{d}{m}\;u+\frac{P}{m}+\alpha \;{\dot{e}}_{r}+\lambda \;\frac{p}{q}\;e_{r}\;{\left(\int_{0}^{t}e_{r}\;dt\right)}^{\frac{p-q}{q}}\right)$$

As $u=u_{smc}=u_{eq}+u_{sw}$, it can be replaced in Equation (21) to obtain Equation (22):

(22) $$\dot{V}\left(s\right)=s\;\dot{s}=s\left(-\frac{b}{m}{\dot{e}}_{r}-\frac{k}{m}e_{r}+\frac{d}{m}\;\epsilon_{ann}+\frac{d}{m}\;\left(u_{eq}+u_{sw}\right))+\frac{P}{m}+\alpha \;{\dot{e}}_{r}+\lambda \;\frac{p}{q}\;e_{r}\;{\left(\int_{0}^{t}e_{r}\;dt\right)}^{\frac{p-q}{q}}\right)$$

By replacing Equation (19) in the expression above, we obtain Equation (23):

(23) $$\begin{array}{cc} \; & \dot{V}\left(s\right)=s(-\frac{b}{m}{\dot{e}}_{r}-\frac{k}{m}e_{r}+\rho -\frac{d}{m}\;k\;sign\left(s\right)+\alpha \;{\dot{e}}_{r}+\lambda \;\frac{p}{q}\;e_{r}\;{\left(\int_{0}^{t}e_{r}\;dt\right)}^{\frac{p-q}{q}}\\ \; & +\frac{b}{m}{\dot{e}}_{r}+\frac{k}{m}e_{r}-\alpha \;{\dot{e}}_{r}-\lambda \;\frac{p}{q}\;e_{r}\;{\left(\int_{0}^{t}e_{r}\;dt\right)}^{\frac{p-q}{q}}\;)\\ \; & =s\;\rho -k\cdot s\cdot sign(s)\\ \; & =s\;\rho -k\left|s\right| \end{array}$$

Therefore, it can be established that to satisfy the condition $\dot{V}\left(s\right)<0$, the switching constant must be $k>\left|\rho \right|$. The conventional use of the sign(s) function often introduces a phenomenon known as the chattering effect, which is characterized by rapid and erratic switching between control actions. In order to mitigate this effect, a common approach is to replace the sign(s) function with a hyperbolic tangent function tanh(βs). The hyperbolic tangent function provides a smooth transition between positive and negative values of *s*, thus reducing abrupt changes in control signals. By introducing a parameter β, the sensitivity of the hyperbolic tangent function can be adjusted to achieve the desired level of smoothing.

## 3. Experimental Results

### 3.1. ANN Training

The ANN training process utilized data recorded from the triangular input signal, which featured an amplitude of 150 V and a period of 1 s. The training dataset consisted of time series data comprising the input voltage and corresponding displacement measurements. Multiple sets of one hysteresis cycle were employed for training, evaluation, and testing, with a partition ratio of 70/20/10, respectively. Detailed specifications are provided in Table 2. The training hardware comprised a Dell Precision 3640 workstation configured with a sampling time of 0.0001 s and parallel calculation enabled across seven cores.

**Figure 5:**
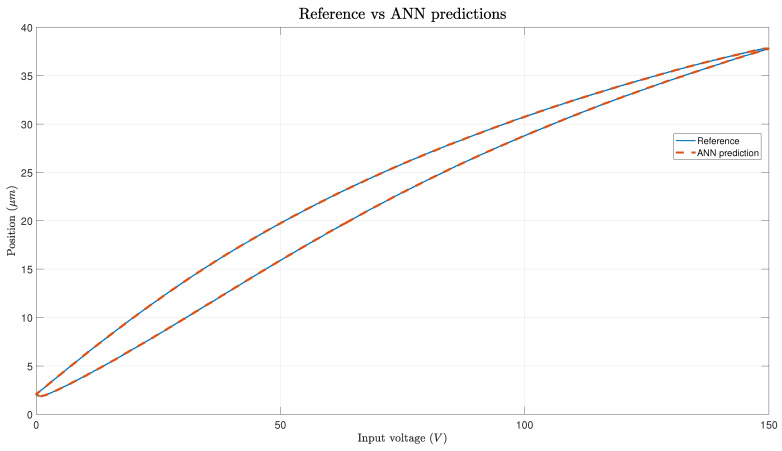
Real data vs. prediction errors.

**Figure 6:**
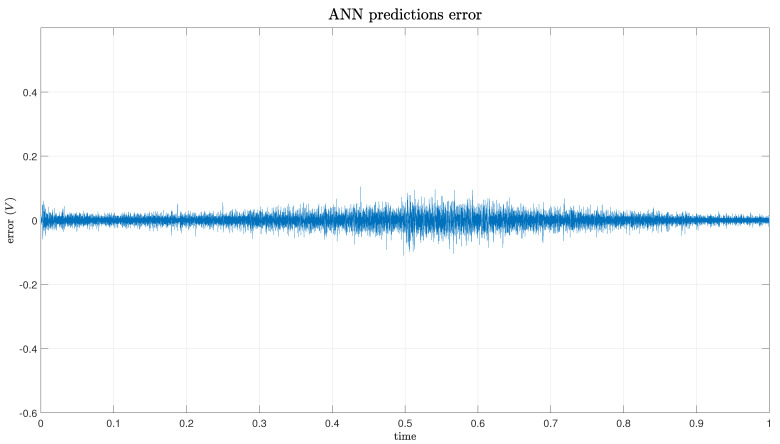
ANN prediction error.

The predictions generated by the ANN exhibit remarkable accuracy for modeling hysteresis, closely aligning with real PEA data, as depicted in Figure 5. The figure depicts both the actual voltage and the predicted voltage over a 1 s cycle. The cycle commences at its peak, descends to the lowest point at the 0.5 s mark, and subsequently ascends back to the peak again. Slight discrepancies are noticeable at the extremes of hysteresis, particularly near the maximum displacement value of 38 V (at 0.5 s), as illustrated in Figure 6. However, these errors are relatively minor, with max absolute values around 0.1 V, as demonstrated in Figure 6; the errors fall within an acceptable range. Excluding these specific areas, the error remains consistently low across the entirety of the hysteresis loop: fluctuating between 0 and 0.05 V.

### 3.2. Reference Tracking

#### 3.2.1. 1 Hz Reference Signals

The PI controller and the ANN-based IFTSMC architectures described in the previous sections were implemented on dSPACE hardware for experimental validation. Two distinct experiments were conducted to evaluate the performance of these controllers. In the first experiment, a 1 Hz triangular reference signal, the same as the training data used for the ANN, was employed. This choice allowed for a direct assessment of the controller’s performance under conditions similar to those encountered during the training phase. Subsequently, a second experiment was conducted utilizing a 1 Hz sinusoidal reference signal. This alternative signal was selected to evaluate the generalization capabilities of the proposed controller beyond the specific signal used for training. By exposing the controller to a different signal than that of the training set, the experiment aimed to assess its ability to adapt and maintain robust performance across varying input conditions.

**Figure 7:**
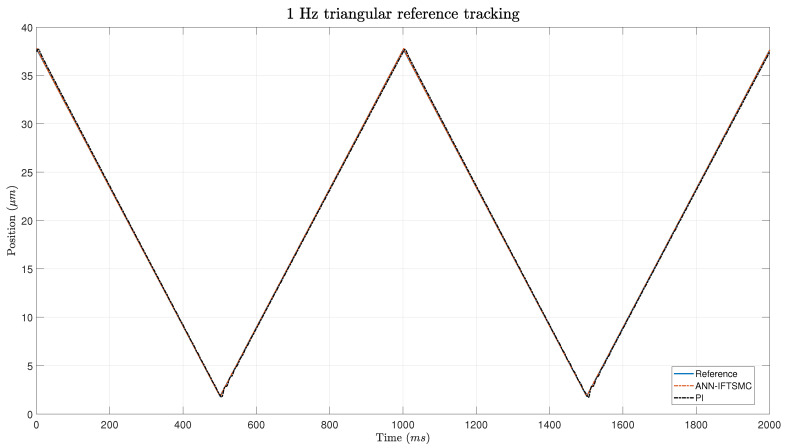
Tracking of a 1 Hz triangular signal for PI- and ANN-based IFTSMC.

**Figure 8:**
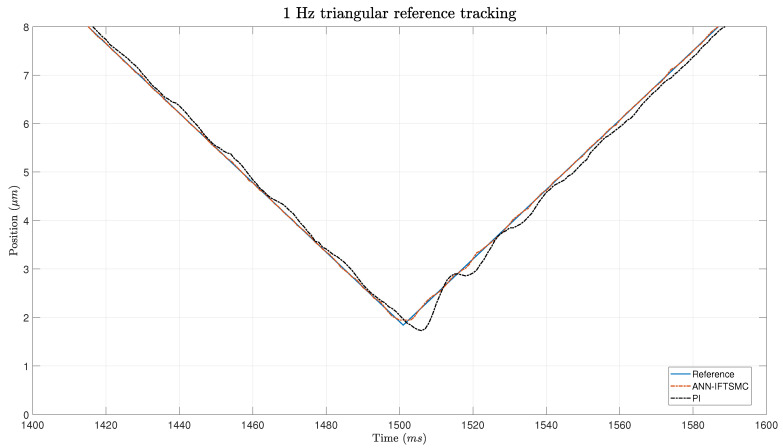
Zoom of the tracking of a 1 Hz triangular signal for PI- and ANN-based IFTSMC.

The parameter selection process was conducted through online tuning techniques. Real-time performance evaluation of the controllers was based on the IAE, which needed to be minimized to optimize system performance. To ensure the safety and stability of the system during operation, each control structure was equipped with several safety features. These included saturation limits to restrict the input voltage within the range of 0–150 V, thus preventing voltage spikes or overloading of the system components. Additionally, anti-wind-up mechanisms were implemented to mitigate the effects of integral action saturation, ensuring stable controller operation even under extreme conditions. Table 3 shows the PI and IFTSMC parameters obtained from online tuning:

As mentioned previously, the first experiment utilized a 1 Hz triangular function as the reference signal. This choice was deliberate in order to introduce complexities characterized by discontinuities, which pose challenges for the controllers in tracking the reference accurately. The triangular waveform has an amplitude spanning nearly the entire range permitted by the PEA, ranging from 1.84 μm to 37.7 μm. To ensure the integrity and longevity of the PEA and other system components, this safety margin was incorporated into the reference signal. This safety margin served to reduce stress on the PEA by preventing it from operating at its maximum capacity, thereby mitigating the risk of mechanical failure or damage due to excessive strain. Figure 7 show the tracking of a 5 Hz triangular reference while Figure 8 shows a more detailed zoom of the areas around the inferior edge.

In Figure 7, both controllers are observed to track the triangular reference signal. However, a notable difference in behavior is apparent between the two controllers. The PI controller exhibits more erratic behavior characterized by small oscillations, which is particularly evident during the transitions between different slopes of the triangular waveform. This behavior is expected due to the presence of discontinuities at these points, which significantly impact the tracking performance of the PI controller.

On the other hand, the ANN-based IFTSMC demonstrates superior overall performance throughout the tracking process. However, it exhibits slightly poorer performance around the edges of the triangular waveform. This can be attributed to the increased complexity of non-linearities present at these points, which pose challenges for the ANN-based controller. Despite this, the ANN-based IFTSMC outperforms the PI controller in terms of various performance metrics. For instance, in terms of the IAE, the ANN-based IFTSMC achieves a value of 0.0259 μm, while the PI controller obtains a value of 0.2934 μm, representing an 11.46-fold improvement in performance. Similarly, the RMSE and RRMSE also demonstrate significant improvements with the ANN-based IFTSMC, exhibiting 6.17-fold and 8.72-fold improvements, respectively, compared to the PI controller.

**Figure 9:**
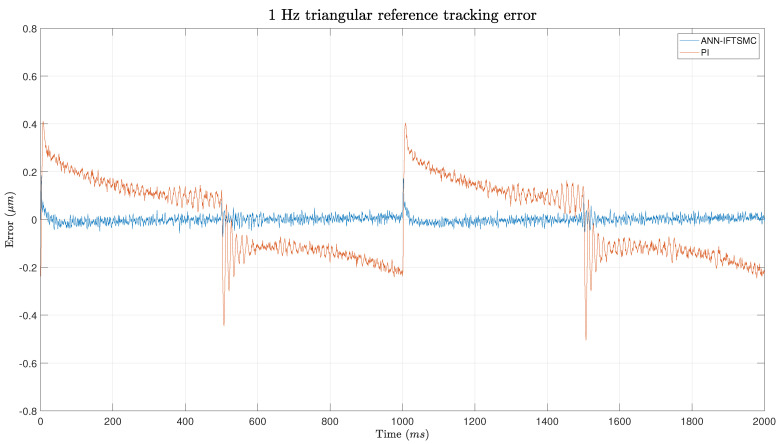
Error of the tracking of a 1 Hz triangular signal for PI- and ANN-based IFTSMC.

Figure 9 depicts the error with respect to the reference signal throughout the experiment and provides further insight into the performance of the two controllers. Consistent with the observations made earlier, the PI controller exhibits oscillatory behavior, which is particularly pronounced around the edges of the triangular waveform. As illustrated in the figure, these oscillations result in significant error spikes, with values exceeding 0.4 μm during the transitions between different slopes. Moreover, the change of sign in the error between positive and negative slopes indicates that the PI controller consistently lags behind the reference signal, which is a characteristic inherent to this controller type.

In contrast, the ANN-based SMC displays a different error profile. At the points of slope change, the ANN-based controller exhibits a small error, which can be attributed to the non-linearities and modeling inaccuracies inherent in the ANN model in this region. Specifically, at the lower point of the slope change, the error spike reaches approximately 1.8 μm, while at the upper point, the error remains much smaller, below 0.1 μm. This discrepancy suggests that the ANN model is more accurate in tracking the upper portion of the waveform compared to the lower portion. Throughout the rest of the path, the error oscillates around the origin, with values consistently below 0.05 μm. Overall, the error analysis provides further evidence of the superior performance of the ANN-based SMC over the PI controller, particularly in terms of error magnitude and consistency.

**Figure 10:**
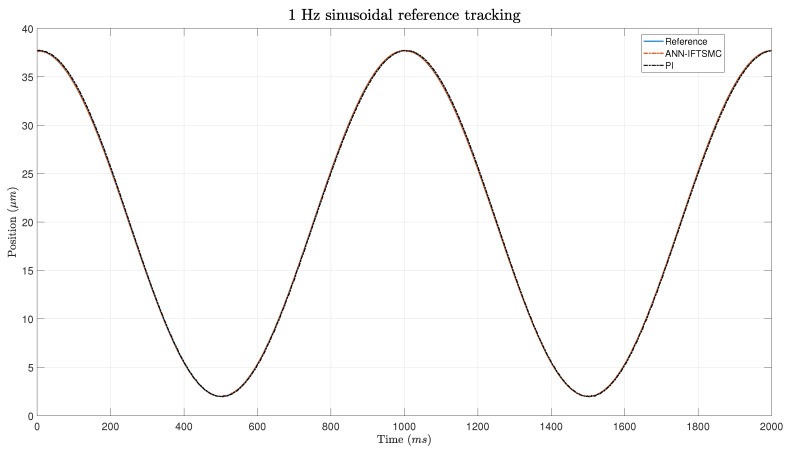
Tracking of a 1 Hz sinusoidal signal for PI- and ANN-based IFTSMC.

**Figure 11:**
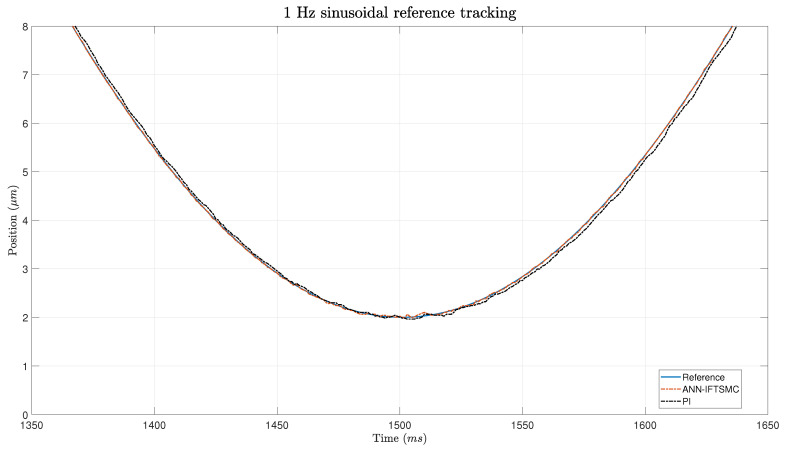
Zoom of the tracking of a 1 Hz sinusoidal signal for PI- and ANN-based IFTSMC.

The experimental evaluation was extended to include soft reference signals with sinusoidal characteristics, with the aim of assessing the controllers’ ability to track more gradual changes and the ANN’s generalization ability. Despite the anticipated improvement in tracking performance, as the sinusoidal edges introduced smoother transitions compared to the triangular trajectories, Figure 10 and the zoomed Figure 11 reveal that the PID controller still struggles to accurately follow these signals. However, it exhibits a slight improvement in response compared to the previous results, indicating a degree of adaptability to the softer input.

In contrast, the ANN-based IFTSMC demonstrates superior error management even when confronted with the softer reference signals. As illustrated in the figure, the ANN-based controller effectively mitigates errors associated with the gradual transitions, maintaining performance levels comparable to those achieved with the sharper triangular signals. Notably, the similarity in amplitudes between the soft and sharp signals facilitates a smooth transition in the slope of the reference trajectory, enabling the ANN-based controller to operate with consistent efficacy across different signal profiles. The experiment revealed a drawback, specifically in the lower portion of the sinusoidal wave trajectory. At this juncture, the ANN-based controller exhibits suboptimal generalization capabilities, leading to a deviation from the reference trajectory. However, this error is swiftly rectified through the action of the switching term, which effectively corrects the deviation and restores the system’s alignment with the desired trajectory. While the ANN demonstrates robust performance across the majority of the sinusoidal wave, its limitations start to become apparent in regions that deviate from the training scenario. Despite this discrepancy, the ANN’s ability to rapidly adapt to and correct errors underscores its effectiveness at maintaining overall trajectory tracking performance, even in the presence of challenging signal dynamics and modeling inaccuracies.

**Figure 12:**
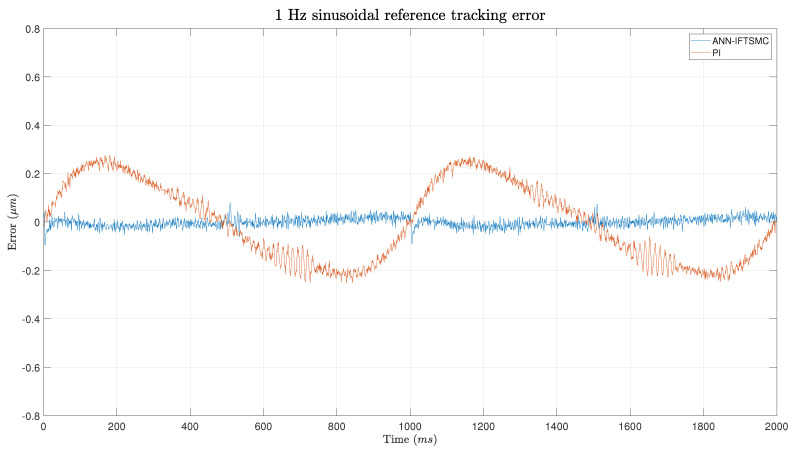
Error of the tracking of a 1 Hz sinusoidal signal for PI- and ANN-based IFTSMC.

Figure 12 provides a visual representation of the observed discrepancy, which is particularly evident in the error performance of the ANN-based IFTSMC controller. Throughout the trajectory, the controller maintains a consistently low error rate, with a minor spike reaching just under 0.1 μm observed at the specified point. However, this error is swiftly corrected, and the controller promptly resumes tracking the reference trajectory with minimal deviation. Conversely, the PI controller exhibits a lower error rate compared to its performance on the triangular signal. The smoother transitions inherent in the sinusoidal wave effectively mitigate the abrupt changes in slope observed in the previous trajectory. Despite this improvement, the metrics once again underscore the superior performance of the ANN-based IFTSMC controller, reaffirming its effectiveness in trajectory tracking tasks. With respect to the IAE, the ANN-based IFTSMC achieves 8.77-times better results than the PI controller, and for both RMSE and RRMSE the performance is 6.9 times better.

In this research, the primary objective was to achieve a reduction in error across the specified trajectories, thereby enhancing overall accuracy. Consequently, the IAE was targeted for reduction through the tuning of corresponding controller gains. Subsequently, performance metrics were calculated over the duration of the reference signals used in the experiments. Table 4 presents a comprehensive comparison of the IAE, RMSE, and RRMSE values obtained for both controllers across the two different signal types: offering insights into their relative effectiveness at error reduction across various scenarios and signal characteristics.

Table 4 presents a comprehensive comparison between the IAE, mean IAE, RMSE, and RRMSE metrics obtained from experiments conducted with both the ANN-based IFTSMC and the PI controller. Across various reference signals, including triangular and sinusoidal patterns, the ANN-based IFTSMC consistently outperformed the PI controller in terms of error reduction and tracking accuracy. Specifically, the IAE values exhibit a significant improvement with the ANN-based IFTSMC, indicating its superior ability to minimize error throughout the trajectory. Additionally, the mean IAE values reflect a substantial reduction, suggesting enhanced performance of the ANN-based IFTSMC in minimizing error over the entire trajectory. The RMSE values further corroborate these findings: demonstrating notably lower error levels with the ANN-based IFTSMC compared to the PI controller. Moreover, the RRMSE values highlight a considerable improvement with the ANN-based IFTSMC, underscoring its effectiveness at reducing error relative to the PI controller. Overall, the metrics affirm the superior performance of the ANN-based IFTSMC in terms of tracking accuracy and error reduction.

#### 3.2.2. 10 Hz Reference Signals

**Figure 13:**
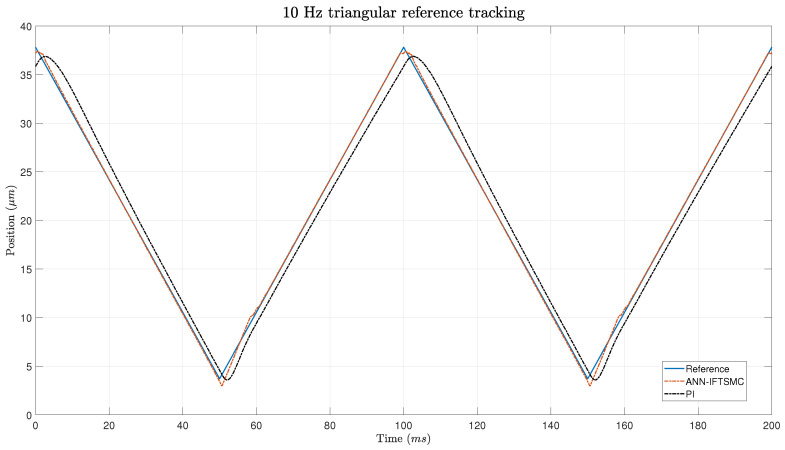
Tracking of a 5 Hz triangular signal for PI- and ANN-based IFTSMC.

To further evaluate the controllers’ capabilities at higher frequencies, a second experiment was conducted at 10 Hz. Lower-frequency tests, such as at 1 Hz, might not fully reveal the limitations of a control system, whereas higher-frequency tests can expose issues like lag and instability. Demonstrating that the IFTSMC maintains its performance at 10 Hz, whereas the PI controller’s performance degrades, highlights the robustness and reliability of the IFTSMC under varying conditions. This is particularly relevant for real-world systems, such as those with vibrating components or electronic circuits, that often operate at higher frequencies. Thus, the 10 Hz experiment underscores the comparative advantage of the IFTSMC, making it more attractive for applications where consistent performance across different frequencies is critical. Figure 13 shows the tracking performance of the controllers at 10 Hz for a triangular signal. It is evident that the IFTSMC controller effectively keeps track of the triangular signal, while the PI controller lags behind the reference, introducing significant error. Although the IFTSMC controller exhibits some degradation in performance at the extremes compared to the 1 Hz experiment, overall it still does a commendable job at maintaining accurate signal tracking. This demonstrates the superior capability of the IFTSMC for handling higher-frequency signals, despite the slight performance dip at the signal’s extremes.

**Figure 14:**
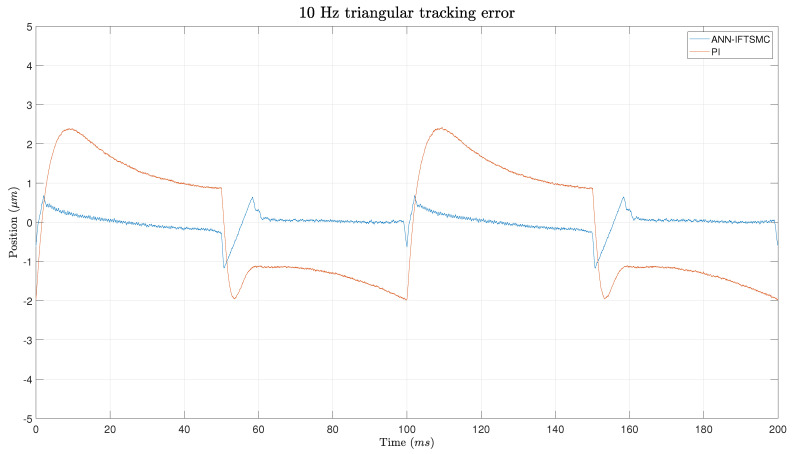
Error of the tracking of a 5 Hz triangular signal for PI- and ANN-based IFTSMC.

Figure 14 shows the error during the experiment. The PI controller exhibited sharp fluctuations, especially around the edges of the experiment, where error measurements spike. Despite these fluctuations, the PI’s method consistently maintains an error above 1 μm throughout the trials, indicating a struggle to achieve lower error values. On the other hand, the IFTSMC method also shows smaller changes around the edges, and it corrects them quickly and converges towards zero error. This highlights the adaptive nature of the IFTSMC method, which efficiently corrects errors and converges towards higher precision; this is especially evident at the experiment’s challenging edges.

**Figure 15:**
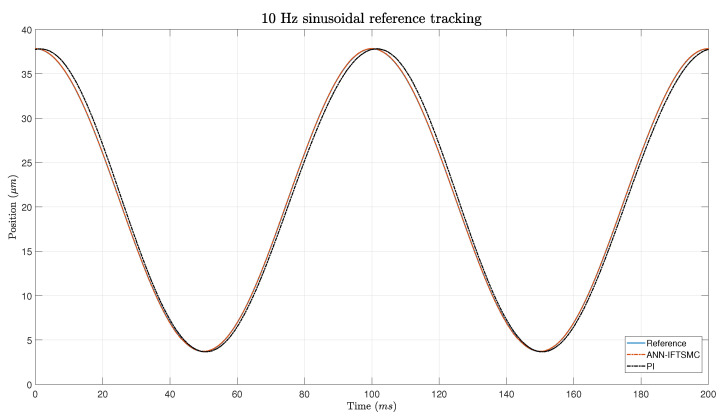
Tracking of a 5 Hz sinusoidal signal for PI- and ANN-based IFTSMC.

In Figure 15, the graph depicts the error measurements of sinusoidal signals at 10 Hz using the IFTSMC and PI methods. The IFTSMC method demonstrates consistent and low error values and maintains them within a narrow range of around 0.05 μm throughout the experiment. This stability is attributed to the absence of sudden changes in the signal, which enables the IFTSMC method to perform well. Conversely, the line representing the PI method shows a delay in response, leading to degraded performance compared to the IFTSMC method. Although the error is slightly smaller than with triangular signals, it remains higher than the IFTSMC’s error due to the PI controller’s delay.

**Figure 16:**
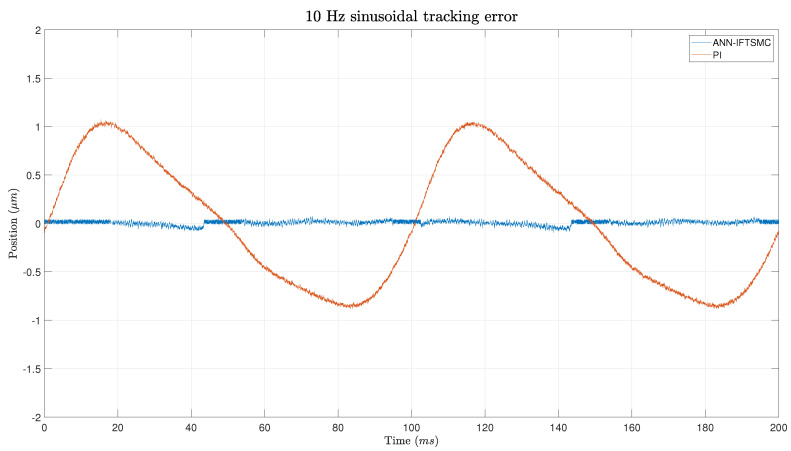
Error of the tracking of a 5 Hz sinusoidal signal for PI- and ANN-based IFTSMC.

Figure 16 illustrates the error measurements of sinusoidal signals at 10 Hz using the same IFTSMC and PI methods. Compared to the 1 Hz scenario, both methods exhibit worse performance due to the increased frequency of the signal. However, the IFTSMC method still manages to maintain relatively low error values, although they are slightly higher than in the 1 Hz case. The lack of sudden changes in the signal contributes to this performance stability. Conversely, the PI method continues to demonstrate a delay in response, resulting in a degradation of performance compared to the IFTSMC method. Despite this, the PI’s error remains slightly smaller than for the triangular signals; this is mainly due to the absence of sudden changes in the signal’s edges.

Table 5 presents error performance indicators for the ANN-based IFTSMC and PI control methods applied to 10 Hz triangular and sinusoidal reference signals. Across all metrics, including IAE, RMSE, and RRMSE, the ANN-based IFTSMC method consistently outperforms the PI method. Notably, for sinusoidal signals, the difference in performance between the two methods is more pronounced, with the ANN-based IFTSMC method demonstrating significantly lower error values. These findings underscore the superior tracking capabilities of the ANN-based IFTSMC method, particularly in scenarios involving sinusoidal reference signals, and highlight its potential for providing more accurate and precise control in such applications. Compared to the metrics from Table 4, the IFTSMC method shows a small increase in all metrics, as expected. Moreover, the increase due to sinusoidal reference tracking is very low, indicating good performance for these type of signals, even with higher frequencies. The PI performance metric shows a large increase in its values. These metrics confirm the poor performance of the PI controller at higher frequencies. In conclusion, although there was a slight deterioration in the performance of the IFTSMC, as expected, the results show that the ANN-based IFTSMC still achieves satisfactory results at higher frequencies. However, the performance of the PI significantly worsened, showing that the proposed controller is more robust and performs better at different frequencies.

## 4. Conclusions

The integration of micro-actuators into industrial environments has revolutionized high-precision positioning, expanding its applications beyond academic domains. Piezoelectric actuators (PEAs) have emerged as indispensable components in various fields and facilitate precise control and manipulation of mechanical systems. Despite their advantages, PEAs also present challenges, such as the necessity to create mathematical models for their non-linear behavior due to hysteresis, creep, and temperature sensitivity. Addressing these challenges is crucial for enhancing the accuracy and reliability of PEA-based systems. In this context, robust controllers offer stability and performance amidst uncertainties and disturbances. This paper proposes an ANN-based IFTSMC controller for PEA positioning, addressing the need for accurate modeling of PEA dynamics.

The recurrent artificial neural network (RNN) has demonstrated remarkable effectiveness in accurately modeling hysteresis behavior. By leveraging its feedback connections, the RNN captures the inherent temporal dynamics in the hysteresis phenomena, achieving precise modeling of the complex non-linear relationships between input variables and output responses. This combination enables the controller to effectively mitigate uncertainties and disturbances, ensuring robust system behavior even in challenging operating conditions. Overall, the utilization of a recurrent ANN in conjunction with robust controllers represents a significant advancement in control system design and offers enhanced accuracy, stability, and resilience in real-world applications.

A Lyapunov stability proof was presented to unveil the theoretical underpinnings of the ANN-based IFTSMC controller. This analysis showed that the controller yields a stable response, contingent upon the IFTSMC satisfying specific conditions dictated by its gains. Subsequently, the experimental phase ensued, wherein the proposed control architectures were implemented and tested. The gains of each framework were tuned by leveraging a criterion based on minimizing the integral of absolute error (minIAE). During the experiments, considerable attention was given to ensuring stability. The objective was successfully met, as no unstable responses were observed. Regarding the controller performance in reference tracking, the ANN-based IFTSMC showed remarkable performance compared to the PI controller. Both controllers achieved better results when tracking sinusoidal references rather than triangular ones. The smooth transitions of the sinusoidal signal facilitate controller operation. Nevertheless, the ANN-based IFTSMC achieved 8- to 9-fold better performance than the PI controller. At slope changes within triangular reference signals, the disparities between the controllers become particularly pronounced. However, during the intervals of straight sections between these slopes, the discrepancies between the two controllers diminish significantly. This observation suggests that, while the controllers may diverge in their responses during rapid changes in the slope, they converge in their performance during relatively steady-state conditions. The performance metrics show 5.6- to 11-fold better performance for the ANN-based IFTSMC on triangular references.

Future research endeavors will explore several options to enhance the performance of the ANN-based IFTSMC. One approach involves employing a more sophisticated ANN architecture, as the current design is relatively shallow. Adopting a deep learning approach could potentially yield improvements, although this would necessitate careful consideration of real-time implementation feasibility due to potentially increased computational demands during training. Additionally, enhancements to the IFTSMC itself are under consideration. While the current gains were optimized through parameter minimization and stability conditions, there is potential for further refinement by implementing adaptive algorithms, such as fuzzy-logic- or neural-network-based approaches. These adaptive algorithms could offer improved adaptability and responsiveness to dynamic system conditions [50,51].

## Figures and Tables

**Table 1:** Thorlabs hardware technical details.

PEA PK4FYC2	Value	Units
Dimensions	7.3 × 7.3 × 7.3	mm
Maximum displacement	38.5	μm
Blocking force	1000	N
Resonant frequency	34	kHz
Maximum error	15	%
Driver Cube KPZ101		
Output driving voltage for PEA	150	V
Input driving voltage	0–10	V
Maximum output bandwidth	1	kHz
Reader Cube KSG101		
Output range	0–10	V
Resolution	1	ηm
Pre-Amplifier AMP002		
Output range	0–2	V

**Table 2:** ANN training details.

Parameter	Value
Data points	106,000
Training/Validation/Test	70/20/10%
Iterations	24,000
Epochs	500
Mini Batch Size	10,000 data points
Initial Learning Rate	0.0001
Validation Frequency	100 iterations
Solver	sgdm
Gradient Threshold Method	absolute-value

**Table 3:** Online parameter tuning values for PI and IFTSMC controllers.

PI	Parameter Value
kp	0.05
ki	1000
IFTSMC	
α	100
λ	20
*K*	50
β	0.1
*p*	2
*q*	3

**Table 4:** 1 Hz signal error performance indicators.

Reference		Triangular	Sinusoidal
IAE [μm]	ANN-based IFTSMC	0.0259	0.0313
	PI	0.2934	0.2864
	Difference	×11.3303	×9.1593
IAE mean [μm]	ANN-based IFTSMC	2.5899 × 10^−5^	1.5637 × 10^−5^
	PI	1.4672 × 10^−4^	1.4322 × 10^−4^
	Difference	×5.6652	×9.1593
RMSE [μm]	ANN-based IFTSMC	0.0256	0.0197
	PI	0.1578	0.1603
	Difference	×6.1684	×8.1464
RRMSE [%]	ANN-based IFTSMC	0.4066	0.4414
	PI	3.5474	3.5961
	Difference	×8.7234	×8.1463

**Table 5:** 10 Hz signal error performance indicators.

Reference		Triangular	Sinusoidal
IAE [μm]	ANN-based IFTSMC	0.0591	0.0187
	PI	0.5623	0.5720
	Difference	×9.5135	×30.5458
RMSE [μm]	ANN-based IFTSMC	0.2376	0.0238
	PI	1.4708	0.6406
	Difference	×6.1893	×26.9595
RRMSE [%]	ANN-based IFTSMC	5.2137	0.5214
	PI	32.2692	14.0568
	Difference	×6.1893	×26.9595

## Data Availability

The data presented in this study are available on request from the corresponding author.
